# Function of Follicular Cytokines: Roles Played during Maturation, Development and Implantation of Embryo

**DOI:** 10.3390/medicina57111251

**Published:** 2021-11-16

**Authors:** Rafał Adamczak, Natalia Ukleja-Sokołowska, Kinga Lis, Mariusz Dubiel

**Affiliations:** 1 Department of Obstetrics and Gynecology, Ludwik Rydygier Collegium Medicum in Bydgoszcz, Nicolaus Copernicus University in Toruń, 85-168 Bydgoszcz, Poland; rafaladamczak@o2.pl (R.A.); dubiel@cm.umk.pl (M.D.); 2 Department of Allergology, Clinical Immunology and Internal Medicine, Ludwik Rydygier Collegium Medicum in Bydgoszcz, Nicolaus Copernicus University in Toruń, 85-168 Bydgoszcz, Poland; kinga.lis@cm.umk.pl

**Keywords:** cytokines, IL-8, G-CSF, IFN-γ, infertility, follicular fluid, IVF, in vitro fertilisation, follicular phase

## Abstract

A balance within the immune system is necessary for the proper development of ovarian follicles. Numerous cytokines were detected in follicular fluid, the role of which in reproductive physiology seems crucial. They influence the development and maturation of the follicle, ovulation, and corpus luteum formation, as well as embryo implantation and maintenance of pregnancy. The analysis of follicular fluid requires its collection by puncturing of the ovary, which is usually executed in connection with various gynaecological procedures. When interpreting such test results, clinical indications for a given procedure and the method of patient preparation should be taken into account. This review revealed the results of currently available studies on the concentration of pro-inflammatory cytokines in follicular fluid in various forms of infertility. Additionally, it presented cytokines, whose concentration has a significant impact on the size of ovarian follicles, their number, the effectiveness of in vitro fertilisation, development of the embryo, and chances of correct implantation. Despite the many recent publications, the knowledge of follicular fluid immunology in the context of reproductive pathology is superficial and further research is required to extensively understand the roles of individual cytokines in reproductive pathology. In the future, this knowledge may enable patients’ individual qualifications to individual methods of infertility treatment, as well as the possible adjustment of the treatment regimen to the patient’s immune profile.

## 1. Introduction

A balance within the immune system is necessary for the proper development of follicles. Cytokines promote proper oocyte maturation, timely rupture of follicles, and neoangiogenesis, indirectly contributing to the supply of oxygen, nutrients, and substrates for subsequent steroidogenesis [1].

The exogenous administration of gonadotropins during in vitro fertilisation (IVF) or intracytoplasmic sperm injection (ICSI) can significantly affect the endogenous follicular regulation system and oocyte quality. The analysis of the cytokine and hormonal profile of follicular fluid (FF) in natural and stimulated physiological cycles is crucial in the assessment of its role in follicle development [2].

The central role of cytokines suggests that any modulation during follicle development and oocyte maturation may have a significant impact on the development of physiological conditions for fertilisation. Expanding the knowledge on the role of various cytokines in particular phases of the cycle and follicular maturation may ultimately help in establishing an individual approach to the fertility treatment.

## 2. Immunology of Folliculogenesis

Cytokines were initially closely associated with the immune system as an important mediator of the immune response. These proteins can stimulate or inhibit cell growth, regulate cell differentiation, induce cell chemotaxis, and modulate expressions of other cytokines [3].

Now, it is known, however, that cytokines are synthesised in a wide range of different types of non-immune cells, including normal ovarian cells, where they promote follicular growth processes, steroidogenesis, recruitment, and activation of leukocytes necessary for ovulation and tissue remodelling during ovulation, luteinisation, and luteolysis [3].

Folliculogenesis can be divided into two phases. The first, preantral phase, is gonadotropin independent and is characterised by the growth and differentiation of oocytes. There are several cytokines involved in the primary activation of follicles and their transition from primary to secondary follicles, including the leukaemia inhibitory factor (LIF), basic fibroblast growth factor (b-FGF), stem cell factor (SCF), and bone morphogenic protein 4 (BMP 4). Additionally, in this phase, the oocyte-derived platelet growth factor (PDGF) and b-FGF promote vesicle activation and increase granulosa cell SCF (GC SCF) expression and secretion, which promotes oocyte growth. Oocyte-derived stromal derived factor 1a (SDF 1a) is thought to act in an autocrine/paracrine fashion to inhibit follicle activation. Further, several other factors regulate the primary follicle passage, such as keratinocyte growth factor (KGF), BMP-4, and BMP-7, which are secreted by stromal cells surrounding the primary follicle. Adjacent follicles produce cytokines that can inhibit activation of follicles, such as the anti-Müllerian hormone (AMH), a member of the transforming growth factor b (TGF-b) family [4,5,6,7,8,9,10].

The second phase of follicle development, called antral or gonadotropin-dependent, is mainly characterised by follicular growth caused by a physiological increase in luteinising hormone (LH) levels that end in ovulation. An increase in LH induces expressions of the monocyte chemotactic protein 1 (MCP 1) and interleukin 8 (IL-8) gene by ovarian stromal cells and granulosa-lutein cells, resulting in an influx of monocytes and neutrophils into the preovulatory follicle. These cells are then activated and secrete further mediators, thus promoting tissue degradation through the action of matrix metalloproteinases as well as vascular changes involved in follicle rupture. In this phase, the volume of FF increases, which allows the aspiration and assessment of the concentration of its individual biochemical and immunological properties [11,12,13].

## 3. Cytokines in Follicular Fluid

Numerous cytokines were detected in the FF, the role of which in reproductive physiology seems crucial. They influence the development and maturation of the follicle, ovulation, and formation of the corpus luteum, as well as in the embryo implantation and maintenance of pregnancy [14].

The FF analysis requires its collection by ovarian puncture. Consequently, knowledge about the cytokine composition of FF concerns mainly animal models, and human studies usually include the analysis of fluid collected during various types of gynaecological procedures. When interpreting such test results, the clinical indications for a given procedure as well as the method of patient preparation should be taken into account. The summary of cytokines found in FF along with the women’s ages and stated reasons for infertility is presented in Table 1.

An extremely interesting study by Piccinni et al. included 83 women with regular menstruation who needed ovarian tissue cryopreservation (OTC) due to the need of treating a malignant neoplasm that did not involve the reproductive organ. Participating women were divided into two groups depending on their age (10–29 and 30–40 years). The cytokine analysis was performed using the Bioplex 200 multiplex test (Bio-Rad Laboratories, Hercules, CA, USA) to assess the concentration of IL-1β, IL 1 receptor antagonist (IL-1Ra), IL-2, IL-4, IL-5, IL-6, IL-7, IL-8, IL-9, IL-10, IL-12, IL-13, IL-15, IL-17, interferon gamma (IFN-γ), tumour necrosis factor alpha (TNF-α), G-CSF, granulocyte-macrophage colony-stimulating factor (GM-CSF), vascular endothelial growth factor (VEGF), PDGF, fibroblast growth factor (FGF), interferon gamma-induced protein 10 (IP-10), MCP-1, regulated on activation, normal T cell expressed and secreted (RANTES), eotaxin, macrophage inflammatory protein 1 alpha (MIP-1α), and macrophage inflammatory protein 1 beta (MIP-1β). All 27 tested cytokines were found in the tested FF. The conclusions from the analysis proved to be exceptionally interesting. First, the cycle phase (luteal vs. follicular) did not affect the concentration of hormones nor the cytokines in FF. However, a relationship was observed between the age of the patients and concentration of individual cytokines. Low levels of IL-5 were observed in younger women, the same being absent in women over 30 years of age. In the group under 30 years, concentrations of IL-1Ra, IL-5, IL-8, eotaxin, and RANTES were significantly higher. Moreover, concentrations of IL-2, IL-4, IL-5, IL-10, IL-7, IL-9, IL-12, IL-15, G-CSF, IFN-γ, MCP1, and RANTES in FF were significantly statistically higher with the increasing follicle diameter, while the PDGF and IP-10 concentrations decreased under the same conditions [15]. This study was of great importance as it concerned natural, non-induced ovulation in women with a normal menstrual cycle. On the other hand, it also concerned women struggling with malignant neoplasms of various etiologies, which may also potentially disrupt the cytokine profile of patients [19].

An extremely interesting study was published by Sarapik et al., who presented an analysis of pro- (IL-1β, IL-6, IL-18, IFN-γ, IFN-α, TNF-α, IL-12, and IL-23) and anti-inflammatory cytokines (G-CSF), in addition to which selected chemokines (MIP-1α, MIP-1β, MCP-1, RANTES, and IL-8) in FF were collected from women undergoing IVF. Higher levels of MIP-1α were found in women diagnosed with polycystic ovarian syndrome (PCOS), and the higher levels of IL-23, INF-γ and TNF-α discovered were characteristic of endometriosis while lower levels of IL-β and INF-α were correlated with tubal infertility. Low IL-18 concentration was characteristic of unexplained infertility. Interestingly, a higher concentration of IL-12 correlated with the successful fertilisation of the ovum and development of an embryo, while higher concentrations of IL-18, IL-8, and MIP-1β were associated with the patient’s successful IVF, defined as pregnancy diagnosis in ultrasound examination in 6–7 weeks after IVF [16].

Depending on environmental conditions, IL-18 can stimulate an immune response dependent on both Th 1 and Th 2 lymphocytes. It is important in the process of folliculogenesis and ovulation, inter alia, by inducing cytokines, such as IL-1β, TNF-α, and IFN-γ [18]. Gutman et al. found that the concentration of IL-18, both in the blood serum and FF, positively correlated with the number of oocytes collected from patients qualified for IVF [20]. According to some studies, the role of IL-18 as well as IL-12 and IL-15 are particularly important in the context of embryo implantation. The overexpression of IL-18 may lead to endometrial cytotoxicity by recruiting the uterus natural killer (uNK) cells, but this process is controlled by a number of mediators that under physiological conditions, allowing for proper embryo implantation and angiogenesis. The tumour necrosis factor-like weak inducer of apoptosis (TWEAK) plays a crucial role as it does not affect the expression of IL-18, but it controls the IL-18-dependent cytotoxicity of uNK cells [21,22].

uNK cell-dependent processes exert direct or indirect—both positive and negative—control over the early stages of implantation. These cells secrete a number of cytokines significant for the proper immune regulation, angiogenesis, and development of the placenta which, in turn, allows for pregnancy [23].

Using the multiplex test (Luminex), Lédée et al. assessed the concentrations of 28 cytokines and chemokines in FF collected from 132 women subjected to IVF-ICSI. They found that a significantly higher concentration of IL-2 and INF-gamma was present in the FF of embryos that underwent early cleavage. The presence of CCL5 chemokine was characteristic for high-quality embryos, and IL-12 was significantly higher in highly fragmented embryos. The concentration of granulocyte colony-stimulating factor (G-CSF) in FF correlated with the implantation potential of an embryo. Interestingly, the FF concentration of G-CSF was significantly lower in patients over 36 years old compared with patients <30 years old [17].

Another key aspect of cytokines in FF is comparing their concentration in natural vs. stimulated cycles. An interesting study was published in 2014, in which Bersinger et al. compared the concentration of the chosen cytokines in the serum and FF of 37 modified natural (NC-IVF) and 39 gonadotropin-stimulated controlled-IVF (c-IVF) cycles. Patients undergoing NC-IVF did not receive any medical intervention except for the administration of human chorionic gonadotropin (hCG) to induce ovulation. IL-6, IL-8, IL-10, IL-18, MCP-1, VEGF, and LIF exhibited notably higher median concentrations in the FF than in the serum. The median concentrations of all the aforementioned selected cytokines with a potential for ovarian production were found to be higher in the patients who underwent c-IVF than in those under NC-IVF treatment. The authors unfortunately did not compare the outcome of the INF procedure in relation to the cytokine level [24].

There were studies in the past that have proven that naturally matured oocytes have higher implantation potential than those from conventionally gonadotropin-stimulated (c-IVF) follicles [25,26].

Premature ovarian insufficiency (POI) is the decline of ovarian function prior to age 40. Liu at al., who published an interesting study in 2020, included 35 women with bPOI (defined as women whose ages were <38 years, with serum FSH > 10 IU/L, and AMH ≤ 1.1 ng/mL) and 37 controls with normal ovarian function. All women were undergoing their first IVF/ ICSI cycle. In FF, significantly elevated levels of chemokines MIP-1α, CXCL8, IP-10, eotaxin-1, and growth factors VEGF-D, BDNF, LIF, and bFGF were found in bPOI patients compared with controls. Conversely, RANTES manifested an opposite trend with reduced levels among bPOI patients. All these were significantly correlated with ovarian reserve [27].

Another similar study by Bouet et al. compared 28 patients with ovarian ageing (diminished ovarian reserve–DOR) and 29 controls with a normal ovarian reserve (NOR). The platelet-derived growth factor-BB (PDGF-BB) was the only cytokine with a notably lower concentration in the DOR group than in the NOR group [28].

IFN-α is a family of extracellular signalling proteins with proven antiviral, antiproliferative, and immunomodulatory effects. Interferon type II IFN-γ is another pro-inflammatory cytokine associated with the development of various autoimmune diseases [29,30].

IFN-γ has a complex effect on follicle maturation, fertilisation, and trophoblast maturation. The characteristic increase in IFN-γ levels in patients with endometriosis, not only in FF but also in the blood serum, may reflect the efforts of the immune system to overcome the inhibition of apoptosis and reduce cell proliferation in endometriosis [31]. Moreover, a temporary increase in IFN-γ appears to be necessary for ovulation. The levels of IFN-γ that exceed normal physiological concentrations may inhibit ovulation and contribute to early pregnancy loss as they are toxic to the embryo at the early (2-cell) and late stage (18–20 weeks of pregnancy). However, it is essential at very early stages after conception. In the 2-cell phase, the zygote secretes low levels of TNF-a and IFN-γ, possibly to induce local inflammation in the endometrium prior to implantation. Moreover, IFN-γ reduces sperm motility [32].

IL-12 regulates the cellular immune response. The p40 subunit of IL-12 is necessary for recruitment and activation of many types of inflammatory cells [33]. TNF-α, an acute phase protein, is one of the most important proteins involved in the immune response following pathogen exposure. An extremely interesting study by Wang et al. analysed concentrations of IL-6, IL-10, IL-13, and TNF-α in the peritoneal fluid collected from 55 patients with infertility and endometriosis. A significantly higher concentration of these cytokines was found in the study group as compared with the control group consisting of 30 healthy women, in whom both endometriosis and infertility were excluded [34].

IL-8 is a neutrophil-specific factor involved in inflammatory processes and angiogenesis [35]. Malhotra et al. found increased levels of IL-8 and leptin in peritoneal fluid collected from 58 women treated laparoscopically for endometriosis compared with the group of 28 women who underwent tubal ligation. This confirms inflammation in the peritoneum results from endometriosis [36].

An LF et al. found that serum concentrations of IL-2, IL-4, IL-6, IL-8, IL-21, TNF-α and IFN-γ were higher in women with unexplained infertility, and the concentration of IL-2 -21 correlated with the presence of autoantibodies [37].

In 2020, Kuang et al. published an analysis of serum cytokine concentration of 78 infertile women, 49 of whom had PCOS, and infertility was caused by other reasons in 29 others. All patients were to be treated for infertility by IVF or Intracytoplasmic Sperm Injection-Embryo Transfer (ICSI-ET). In the PCOS group, the concentrations of IL-17a, IL-1Ra, and IL-6 were statistically and significantly higher than in the group where PCOS was excluded, indicating a subclinical inflammation that occurs in patients over a long period of time [38].

It is worth emphasising that there are many causes of subclinical inflammation that may persist in patients for a long time, causing a similar cytokine profile as in the above-mentioned diseases. For example, in atopic diseases such as bronchial asthma, there is increased activity of pro-inflammatory cytokines, including IL-6 and TNF-α, which in turn promotes a NK-cell and neutrophil-mediated response [39,40].

Moreover, PCOS often occurs in patients who meet the criteria of metabolic syndrome. Being overweight and obese also cause chronic, low-severity inflammation in the body [41]. The cytokine profile is somewhat similar to that seen in some forms of infertility. Among others, in obese patients, an increase in concentrations of IL-1, IL-6, and TNF-α is observed [42]. Chronic inflammation, including that associated with increased levels of, e.g., IL-6, also promotes the development of cancer [43,44]. The examples given depict how complex the homeostasis of the immune system is and how numerous processes can influence a patient’s cytokine profile, rendering it difficult to interpret the obtained test results in the context of determining the exact causes of reproductive disorders.

The summary of FF cytokines and correlation of their concentration with follicular and embryo development are presented in Table 2.

## 4. Practical Aspects and Unanswered Questions

The composition of the FF reflects the exchanges between the oocyte and its microenvironment. It has been investigated to determine the metabolic pathways involved in various ovarian disorders as well as its immunological potential [28].

It is extremely difficult to assess the interactions between cytokines of FF and blood serum of patients with infertilities of different origin. It is unfortunately a hard task. The cytokines mentioned play important roles in many other conditions, the interactions of which are complex and require in-depth analyses. For instance, we are aware that in asthma, IL-4 and IL-5 hold crucial roles, whereas in inflammatory bowel disease and rheumatoid arthritis, TNF-α levels are heightened as a result of chronic inflammation. Further, in chronic obstructive pulmonary disease, cigarette smoke promotes IL-8 production [45,46,47]. In the current COVID-19 pandemic, IL-6 and IL-8 are some of the relevant markers of the cytokine storm and may help in predicting the severity of inflammation [48]. These are only few examples of diseases where there are well-described biological agents to help treat them, with quality-of-life changing effects.

One of the most compelling questions would be in asking if modifying the cytokine profile of the patient would enable increasing the chance of successful outcomes of fertility procedures. In general, the treatment of the underlying reason of cytokine profile disruption is beneficial and may reverse the diseases’ negative effects on fertilisation and pregnancy outcomes. A good example of this is PCOS, in which insulin resistance therapy, body weight reduction, and lifestyle changes, as well as pharmacological treatments, for example, metformin, can have a beneficial effect on the leptin levels and cytokine profile of patients [49,50,51].

Endometriosis is a highly inflammatory, proangiogenic process, which impairs fertility by influencing the oocyte and embryo quality along with the transport and implementation of the embryo in the reproductive track. It is gathered with a wide range of proinflammatory cytokines, which may potentially be a target for treatment. The treatment of the disease consists of a wide range of methods, including surgical treatment, preferably laparoscopy, pharmacological methods, or both. The first-line medical treatment remains suppressive and not curative, including non-steroidal anti-inflammatory drugs and combined oral contraceptives (COCs) or progestins. Numerous anti-angiogenic molecules have been evaluated in the treatment of endometriosis and have shown promising results in terms of the regression of hypervascularised lesions, but more studies are necessary before these drugs can be routinely applied [52].

The suggested role of inflammation and immune cell deregulation in infertility associated with endometriosis imparts hope for future immunomodulatory treatments in endometriosis combined with infertility. A very interesting metanalysis on the subject was published in 2021, in which the authors found that most studies concentrate around the use of TNF-α antagonists; although there are some promising results, they still predominantly include animal models (baboons or rats) [53].

In an available 21-participant human study, there were no evidence of an effect of infliximab (anti-TNF-α) on pelvic pain reduction or the use of pain killers in endometriosis patients. Similarly, there was no proof of an increase in adverse events in the infliximab group but also no evidence of clinical benefits of infliximab for endometriotic lesions, dysmenorrhoea, dyspareunia, or pelvic tenderness. Its influence on fertility was also not researched in the cited study [54].

Further research is required to evaluate the biological agents in different conditions gathered with infertility. The biological agents administered in women during their reproductive age and pregnancy generated concerns about the safety of the foetus. The bioethical issue is also of interest when designing studies on this group of patients. In 2020, Goralczyk at al. published a review article on the safety of biological and targeted synthetic disease-modifying antirheumatic drugs (bDMARDs and tsDMARDs) during conception, pregnancy, and lactation. The authors discovered that there are no solid data confirming the negative effect of TNF-αi, rituximab, and abatacept on fertility and an increased number of complications due to paternal exposure. The available studies suggest the safety of bDMARDs and tsDMARDs in pregnant women [55]. There is currently a clinical trial open on the safety of benralizumab (anti-IL5 receptor antibody) in pregnancy in women with severe asthma [56]. The first case reports of successful pregnancy in unexplained infertility in women, who suffered from severe, eosinophilic asthma, who became pregnant after successful mepolizumab (anti-IL5) treatment also exists. Although there are no recommendations or large studies to support the thesis, it might suggest that treating underlying health conditions and correcting immunological imbalance can have a positive effect on fertility [57].

There are also other aspects of described FF cytokine levels that would require further analysis and interpretation. The FF is not a biomaterial routinely acquired from the patient; rather, it is generally received as a part of fertility treatment procedures before IVF or during fertility preservation in patients, requiring intensive treatments with the potential of damaging future child-bearing chances. The healthy human FF is not routinely evaluated due to lack of indications of puncture of ovary under normal circumstances, which can influence the results of published studies. Are high levels of different cytokines acquired in cited studies a physiological phenomenon or a result of the current patients’ health situation? Is a protocol for fertility treatment an issue in the patients’ cytokine profiles? IVF procedures also have other aspects that have to be taken into account. In many countries, gestational surrogacy is illegal. Women going through the procedure in countries where it is possible are underdiagnosed from an immunological point of view. There is proof that the placental material of women after IVF with donor eggs is characterised by a complex immune response in sites of tight contact between maternal and foetal tissues. The impaired immune tolerance of the donor genetic material is associated with, for example, an increase in the number of HLA-DR positive cells as well as the development of areas of chronic inflammation in perivascular regions [58]. More studies are necessary to evaluate the cytokine profile of these women.

## 5. Conclusions

Pro-inflammatory cytokines are crucial in the maturation process of the ovarian follicle, in addition to the process of embryo implantation. Immune imbalances have a negative impact on the prognosis of the effectiveness of IVF and possibly on natural fertilisation. The role of IL-12 in FF seems to be of particular interest, as—according to some studies—its higher concentration is correlated with effective fertilisation of an ovum and the development of an embryo. According to other researchers, the cytokine profile of IL-1Ra, IL-5, IL-8, eotaxin, and RANTES occurs in young patients at the peak of fertility, and the concentrations of these interleukins decrease with age. Interestingly, according to certain studies, IL-18, both in blood serum and FF, positively correlates with the number of oocytes collected from patients qualified for IVF. These and other observations certainly require an in-depth analysis and further studies based on carefully selected populations to allow for an accurate interpretation of results and consider how the identified cytokine profile influences the prognosis in infertility treatments.

## Figures and Tables

**Table 1:** Cytokines of the follicular fluid and their correlation with age of women and different reasons for infertility.

Study name	Age (<30)	Age (>36)	PCOS	Endometriosis	Tubal Infertility	Unexplained Infertility
Piccinni MP et al., 2021 [15]	↑ IL-1Ra, IL-5, IL-8, eotaxin, RANTES ↓ IL-5					
Sarapik et al., 2011 [16]			↑ MIP-1α	↑ IL-23, INF-γ, TNF-α	↓ IL-β and INF-α	↓ IL-18
Lédée N et al., 2008 [17]	↑ G-CSF	↓ G-CSF				
Nishida M. et al., 2005 [18]				↑ IFN-γ		

**Table 2:** Cytokines of follicular fluid and correlation of their concentration with follicular and embryo development during fertility procedures.

Study Name	Increased Follicle Diameter during Puncture	Increased Number of Embryos that Underwent Early Cleavage	Increased Number of Oocytes Collected from Patients Qualified for IVF	Increased Number of High-Quality Embryos	The Number of Highly Fragmented Embryos	The Rate of Successful Fertilisation of the Ovum and Development of an Embryo	Chance of Successful in Vitro Fertilisation (Pregnancy in 6–7 Weeks after IVF)
Piccinni MP et al., 2021 [15]	↑ IL-2, IL-4, IL-5, IL-10, IL-7, IL-9, IL-12, IL-15, G-CSF, IFN-γ, MCP1, and RANTES ↓ PDGF, IP-10						
Sarapik et al., 2011 [16]						↑ IL-12	↑ IL-18, IL-8, MIP-1β
Gutman et al., 2004 [20]			↑ IL-18				
Lédée et al., 2008 [17]		↑ IL-2, INF-gamma		↑ CCL5 chemokine	IL-12		

## Data Availability

Not applicable.

## References

[1] Baskind N.E., Orsi N.M., Sharma V. (2014). Follicular-phase Ovarian Follicular Fluid and Plasma Cytokine Profiling of Natural Cycle In Vitro Fertilization Patients. Fertil. Steril..

[2] Santos M.A., Kuijk E.W., Macklon N.S. (2010). The impact of ovarian stimulation for IVF on the developing embryo. Reproduction.

[3] Büscher U., Chen F.C., Kentenich H., Schmiady H. (1999). Cytokines in the Follicular Fluid of Stimulated and Non-stimulated Human Ovaries: Is Ovulation a Suppressed Inflammatory Reaction?. Hum. Reprod..

[4] Nilsson E., Parrott J.A., Skinner M.K. (2001). Basic Fibroblast Growth Factor Induces Primordial Follicle Development and Initiates Folliculogenesis. Mol. Cell Endocrinol..

[5] Parrott J.A., Skinner M.K. (1999). Kit-ligand/Stem Cell Factor Induces Primordial Follicle Development and Initiates Folliculogenesis. Endocrinology.

[6] Nilsson E.E., Skinner M.K. (2003). Bone Morphogenetic Protein-4 Acts as an Ovarian Follicle Survival Factor and Promotes Primordial Follicle Development. Biol. Reprod..

[7] Holt J.E., Jackson A., Roman S.D., Aitken R.J., Koopman P., McLaughlin E.A. (2006). CXCR4/SDF1 Interaction Inhibits the Primordial to Primary Follicle Transition in the Neonatal Mouse Ovary. Dev. Biol..

[8] Kezele P., Nilsson E.E., Skinner M.K. (2005). Keratinocyte Growth Factor Acts as a Mesenchymal Factor that Promotes Ovarian Primordial to Primary Follicle Transition. Biol. Reprod..

[9] Durlinger A.L., Kramer P., Karels B., de Jong F.H., Uilenbroek J.T., Grootegoed J.A., Themmen A.P. (1999). Control of Primordial Follicle Recruitment by Anti-Müllerian Hormone in the Mouse Ovary. Endocrinology.

[10] Carlsson I.B., Scott J.E., Visser J.A., Ritvos O., Themmen A.P., Hovatta O. (2006). Anti-Müllerian Hormone Inhibits Initiation of Growth of Human Primordial Ovarian Follicles In Vitro. Hum. Reprod..

[11] Gougeon A. (1996). Regulation of Ovarian Follicular Development in Primates: Facts and Hypotheses. Endocr. Rev..

[12] Brannstrom M., Pascoe V., Norman R.J., McClure N. (1994). Localization of Leukocyte Subsets in the Follicle Wall and in the Corpus Luteum Throughout the Human Menstrual Cycle. Fertil. Steril..

[13] Bukulmez O., Arici A. (2000). Leukocytes in Ovarian Function. Hum. Reprod. Update.

[14] Stewart C.L., Kaspar P., Brunet L.J., Bhatt H., Gadi I., Köntgen F., Abbondanzo S.J. (1992). Blastocyst Implantation Depends on Maternal Expression of Leukaemia Inhibitory Factor. Nature.

[15] Piccinni M.P., Vicenti R., Logiodice F., Fabbri R., Kullolli O., Pallecchi M., Paradisi R., Danza G., Macciocca M., Lombardelli L. (2021). Description of the Follicular Fluid Cytokine and Hormone Profiles in Human Physiological Natural Cycles. J. Clin. Endocrinol. Metab..

[16] Sarapik A., Velthut A., Haller-Kikkatalo K., Faure G.C., Béné M.C., de Carvalho Bittencourt M., Massin F., Uibo R., Salumets A. (2012). Follicular Proinflammatory Cytokines and Chemokines as Markers of IVF Success. Clin. Dev. Immunol..

[17] Lédée N., Lombroso R., Lombardelli L., Selva J., Dubanchet S., Chaouat G., Frankenne F., Foidart J.M., Maggi E., Romagnani S. (2008). Cytokines and Chemokines in Follicular Fluids and Potential of the Corresponding Embryo: The Role of Granulocyte Colony-stimulating Factor. Hum. Reprod..

[18] Nakanishi K., Yoshimoto T., Tsutsui H., Okamura H. (2001). Interleukin-18 is a Unique Cytokine that Stimulates Both Th1 and Th2 Responses Depending on its Cytokine Milieu. Cytokine Growth Factor Rev..

[19] Lippitz B.E. (2013). Cytokine Patterns in Patients with Cancer: A Systematic Review. Lancet Oncol..

[20] Gutman G., Soussan-Gutman L., Malcov M., Lessing J.B., Amit A., Azem F. (2004). Interleukin-18 is High in the Serum of IVF Pregnancies with Ovarian Hyperstimulation Syndrome. Am. J. Reprod. Immunol..

[21] Petitbarat M., Rahmati M., Sérazin V., Dubanchet S., Morvan C., Wainer R., de Mazancourt P., Chaouat G., Foidart J.M., Munaut C. (2011). TWEAK Appears as a Modulator of Endometrial IL-18 Related Cytotoxic Activity of Uterine Natural Killers. PLoS ONE.

[22] Lédée-Bataille N., Bonnet-Chea K., Hosny G., Dubanchet S., Frydman R., Chaouat G. (2005). Role of the Endometrial Tripod Interleukin-18, -15, and -12 in Inadequate Uterine Receptivity in Patients with a History of Repeated In Vitro Fertilization-embryo Transfer Failure. Fertil. Steril..

[23] Lédée N., Petitbarat M., Rahmati M., Dubanchet S., Chaouat G., Sandra O., Perrier-d’Hauterive S., Munaut C., Foidart J.M. (2011). New Pre-conception Immune Biomarkers for Clinical Practice: Interleukin-18, Interleukin-15 and TWEAK on the Endometrial Side, G-CSF on the Follicular side. J. Reprod. Immunol..

[24] Bersinger N.A., Kollmann Z., Von Wolff M. (2014). Serum but not Follicular Fluid Cytokine Levels are Increased in Stimulated Versus Natural Cycle IVF: A Multiplexed Assay Study. J. Reprod. Immunol..

[25] Aanesen A., Nygren K.G., Nylund L. (2010). Modified Natural Cycle IVF and Mild IVF: A 10 year Swedish Experience. Reprod. Biomed. Online.

[26] Gordon J.D., DiMattina M., Reh A., Botes A., Celia G., Payson M. (2013). Utilization and Success Rates of Unstimulated In Vitro Fertilization in the United States: An Analysis of the Society for Assisted Reproductive Technology Database. Fertil. Steril..

[27] Liu P., Zhang X., Hu J., Cui L., Zhao S., Jiao X., Qin Y. (2020). Dysregulated Cytokine Profile Associated with Biochemical Premature Ovarian Insufficiency. Am. J. Reprod. Immunol..

[28] Bouet P.E., Boueilh T., de la Barca J.M.C., Boucret L., Blanchard S., Ferré-L’Hotellier V., Jeannin P., Descamps P., Procaccio V., Reynier P. (2020). The Cytokine Profile of Follicular Fluid Changes During Ovarian Ageing. J. Gynecol. Obstet. Hum. Reprod..

[29] Zidovec Lepej S., Vujisic S., Stipoljev F., Mazuran R. (2003). Interferon-alpha-like Biological Activity in Human Seminal Plasma, Follicular Fluid, Embryo Culture Medium, Amniotic Fluid and Fetal Blood. Reprod. Fertil. Dev..

[30] Damsker J.M., Hansen A.M., Caspi R.R. (2010). Th1 and Th17 cells: Adversaries and Collaborators. Ann. N. Y. Acad. Sci..

[31] Nishida M., Nasu K., Ueda T., Fukuda J., Takai N., Miyakawa I. (2005). Endometriotic Cells are Resistant to Interferon-γ-induced Cell Growth Inhibition and Apoptosis: A Possible Mechanism Involved in the Pathogenesis of Endometriosis. Mol. Hum. Reprod..

[32] Vassiliadis S., Relakis K., Papageorgiou A., Athanassakis I. (2005). Endometriosis and Infertility: A Multi-cytokine Imbalance Versus Ovulation, Fertilization and Early Embryo Development. Clin. Dev. Immunol..

[33] Langrish C.L., McKenzie B.S., Wilson N.J., De Waal Malefyt R., Kastelein R.A., Cua D.J. (2004). IL-12 and IL-23: Master Regulators of Innate and Adaptive Immunity. Immunol. Rev..

[34] Wang X.M., Ma Z.Y., Song N. (2018). Inflammatory Cytokines IL-6, IL-10, IL-13, TNF-α and Peritoneal Fluid Flora were Associated with Infertility in Patients with Endometriosis. Eur. Rev. Med. Pharmacol. Sci..

[35] Runesson E., Boström E.K., Janson P.O., Brännström M. (1996). The Human Preovulatory Follicle is a Source of the Chemotactic Cytokine Interleukin-8. Mol. Hum. Reprod..

[36] Malhotra N., Karmakar D., Tripathi V., Luthra K., Kumar S. (2012). Correlation of Angiogenic Cytokines-leptin and IL-8 in Stage, Type and Presentation of Endometriosis. Gynecol. Endocrinol..

[37] An L.F., Zhang X.H., Sun X.T., Zhao L.H., Li S., Wang W.H. (2015). Unexplained Infertility Patients have Increased Serum IL-2, IL-4, IL-6, IL-8, IL-21, TNFα, IFNγ and Increased Tfh/CD4 T Cell Ratio: Increased Tfh and IL-21 Strongly Correlate with Presence of Autoantibodies. Immunol. Investig..

[38] Kuang H., Duan Y., Li D., Xu Y., Ai W., Li W., Wang Y., Liu S., Li M., Liu X. (2020). The Role of Serum Inflammatory Cytokines and Berberine in the Insulin Signaling Pathway Among Women with Polycystic Ovary Syndrome. PLoS ONE.

[39] Adamczak R., Ukleja-Sokołowska N., Bartuzi Z. (2019). Fertility and Allergy: Is There a Correlation?. PHMD.

[40] Juul Gade E., Thomsen S.F., Lindenberg S., Backer V. (2014). Female Asthma has a Negative Effect on Fertility: What is the Connection?. ISRN Allergy.

[41] Gregor M.F., Hotamisligil G.S. (2011). Inflammatory Mechanisms in Obesity. Annu. Rev. Immunol..

[42] Saxena N.K., Sharma D. (2013). Multifaceted Leptin Network: The Molecular Connection Between Obesity and Breast Cancer. J. Mammary Gland Biol. Neoplasia.

[43] Hirano T. (2021). IL-6 in Inflammation, Autoimmunity and Cancer. Int. Immunol..

[44] Kolb R., Sutterwala F.S., Zhang W. (2016). Obesity and Cancer: Inflammation Bridges the Two. Curr. Opin. Pharmacol..

[45] Khatana U.F., Qamar A., Ashfaq M.B. (2021). Infliximab-Associated Acneiform Eruption in a Patient with Inflammatory Bowel Disease. Cureus..

[46] Zhu X., Zhan Y., Gu Y., Huang Q., Wang T., Deng Z., Xie J. (2021). Cigarette Smoke Promotes Interleukin-8 Production in Alveolar Macrophages Through the Reactive Oxygen Species/Stromal Interaction Molecule 1/Ca^2+^ Axis. Front. Physiol..

[47] Wechsler M.E., Ruddy M.K., Pavord I.D., Israel E., Rabe K.F., Ford L.B., Maspero J.F., Abdulai R.M., Hu C.C., Martincova R. (2021). Efficacy and Safety of Itepekimab in Patients with Moderate-to-Severe Asthma. N. Engl. J. Med..

[48] Cabaro S., D’Esposito V., Di Matola T., Sale S., Cennamo M., Terracciano D., Parisi V., Oriente F., Portella G., Beguinot F. (2021). Cytokine Signature and COVID-19 Prediction Models in the Two Waves of Pandemics. Sci. Rep..

[49] Wang J., Gong P., Li C., Pan M., Ding Z., Ge X., Zhu W., Shi B. (2020). Correlation Between Leptin and IFN-γ Involved in Granulosa Cell Apoptosis in PCOS. Gynecol. Endocrinol..

[50] Soldat-Stanković V., Popović-Pejičić S., Stanković S., Prtina A., Malešević G., Bjekić-Macut J., Livadas S., Ognjanović S., Mastorakos G., Micić D. (2021). The Effect of Metformin and Myoinositol on Metabolic Outcomes in Women with Polycystic Ovary Syndrome: Role of Body Mass and Adiponectin in a Randomized Controlled Trial. J. Endocrinol. Investig..

[51] Amani Abkenari S., Safdarian L., Amidi F., Hosseini A., Aryanpour R., Salahi E., Sobhani A. (2021). Metformin Improves Epigenetic Modification Involved in Oocyte Growth and Embryo Development in Polycystic Ovary Syndrome Mice Model. Mol. Reprod. Dev..

[52] Brichant G., Laraki I., Henry L., Munaut C., Nisolle M. (2021). New Therapeutics in Endometriosis: A Review of Hormonal, Non-Hormonal, and Non-Coding RNA Treatments. Int. J. Mol. Sci..

[53] Kolanska K., Alijotas-Reig J., Cohen J., Cheloufi M., Selleret L., d’Argent E., Kayem G., Valverde E.E., Fain O., Bornes M. (2021). Endometriosis with Infertility: A Comprehensive Review on the Role of Immune Deregulation and Immunomodulation Therapy. Am. J. Reprod. Immunol..

[54] Lu D., Song H., Shi G. (2013). Anti-TNF-α Treatment for Pelvic Pain Associated with Endometriosis. Cochrane Database Syst. Rev..

[55] Góralczyk A., Kolossa K., Waszczak-Jeka M., Adamczak R., Jeka S. (2020). The Exposure to Biologic and Targeted Synthetic Disease-modifying Antirheumatic Drugs in Pregnancy and Lactation. Postepy Dermatol. Alergol..

[56] Clinical Trials https://clinicaltrials.gov/ct2/show/NCT03794999.

[57] Ozden G., Pınar Deniz P. (2021). May Mepolizumab Used in Asthma Correct Subfertility?. Ann. Med..

[58] Kogan E.A., Rudenko E.E., Demura T.A., Zharkov N.V., Trifonova N.S., Zhukova E.V., Aleksandrov L.S., Bayanova S.N. (2019). Structural, Immunohistochemical and Molecular Features of Placentas and Placental Sites After In Vitro Fertilization with Donor Eggs (surrogate motherhood). Eur. J. Obstet. Gynecol. Reprod. Biol..

